# Anti-Phosphatidylserine-Prothrombin Antibodies are Associated with Outcome in a TIA Cohort

**DOI:** 10.3389/fneur.2012.00137

**Published:** 2012-09-28

**Authors:** Michael T. Mullen, Steven R. Messé, Scott E. Kasner, Lauren Sansing, M. R. Husain, Gary L. Norman, Zakera Shums, Brett L. Cucchiara

**Affiliations:** ^1^Department of Neurology, University of PennsylvaniaPhiladelphia, PA, USA; ^2^Department of Neurology, University of ConnecticutFarmington, CT, USA; ^3^INOVA Diagnostics, Inc.San Diego, CA, USA

**Keywords:** anticardiolipin, antiphospholipid, biomarker, infarction, thrombosis, transient ischemic attack, aPS/PT antibodies

## Abstract

**Background:** Antiphospholipid antibodies (aPLs) have been associated with thrombosis in the antiphospholipid antibody syndrome (APS) and with atherosclerotic vascular events in patients without APS. We examined the significance of aPLs in transient ischemic attack (TIA). **Patients/methods:** Patients with TIA <48 h from symptom onset were prospectively enrolled. Traditional aPLs, including anticardiolipin and β2-glycoprotein-I (β2GPI), and newer aPLs, including anti-phosphatidylserine/prothrombin (aPS/PT), β2GPI Domain 4/5 and β2GPI Domain 1 were measured. Primary outcome was a composite of stroke or death within 90 days or identification of a high risk stroke mechanism. Secondary outcomes were stroke or death and the presence of clinical/sub-clinical atherosclerosis. **Results:** Over 4.5 years, 167 patients were enrolled. Forty one patients (25%) had the composite endpoint. Antibodies were measured in 158 subjects. aPS/PT IgG antibodies were significantly associated with stroke/death (OR 16.3, 95% CI 2.3–116.7, *p* = 0.005) and were non-significantly associated with the composite endpoint (OR 4.7, 95% CI 0.8–29.2, *p* = 0.10). In multivariate analysis adjusting for ABCD^2^ risk score, aPS/PT IgG remained associated with stroke/death (OR 15.7, 95% CI 2.0–125.6, *p* = 0.009). Other aPLs were not associated with clinical outcome and no association between APLs and atherosclerosis was identified. **Conclusion:** In contrast to other aPLs, aPS/PT IgG antibodies are independently associated with stroke or death in patients with TIA.

## Introduction

Antiphospholipid antibodies (aPLs) are a diverse collection of autoantibodies which are directed at plasma proteins bound to phospholipid membranes. Traditional aPLs include anticardiolipin (aCL) and β-2-glycoprotein-I (β2GPI) antibodies. These antibodies have been associated with thrombosis in the antiphospholipid antibody syndrome (APS), which is characterized by arterial and venous thrombosis as well as recurrent fetal loss (Cohen et al., 2010). Newly defined sub-classes of aPLs, including antibodies to β2GPI Domain 1 (β2GPI D1) and β2GPI Domain 4/5 (β2GPI D4/5) and anti-phosphatidylserine/prothrombin (aPS/PT), have been associated with thrombosis; although there is not yet sufficient evidence to include these antibodies into APS diagnostic criteria (Atsumi et al., 2000; Bertolaccini et al., 2005, 2011; Forastiero et al., 2005; Bizzaro et al., 2007; Sanmarco et al., 2007). Recently, aPLs have also been linked to atherosclerotic vascular disease in subjects without APS (Farsi et al., 1999; Brey et al., 2001; Staub et al., 2003; Veres et al., 2004; Iverson et al., 2006; Franck et al., 2007; Bizzaro et al., 2010). These findings raise the question of whether APL antibodies may be useful as biomarkers of vascular risk in patients without APS.

Patients with transient ischemic attack (TIA) have a high short-term risk of stroke, with estimates up to 15% at 90 days (Kleindorfer et al., 2005; Easton et al., 2009). Reliable identification of TIA patients at highest risk could be used to select patients for aggressive monitoring and therapeutic interventions. Clinical risk scores, such as the ABCD^2^ score, incorporate features of the patient’s history and symptoms to estimate subsequent stroke risk (Johnston et al., 2007). This risk score has a number of important limitations (Cucchiara et al., 2006; Perry et al., 2011). Risk prediction may be enhanced by incorporating results of brain imaging with MRI to assess for infarction and/or vascular imaging to assess for atherosclerotic carotid stenosis, both of which are associated with a higher risk independent of the ABCD^2^ score (Merwick et al., 2010; Giles et al., 2011). Unfortunately, these studies are resource intensive and not always available in the emergency setting. There is a great need to identify other biomarkers which can reliably identify TIA patients who are at high risk of subsequent stroke.

Because aPLs are potentially associated with thrombosis and atherosclerosis, they may function as biomarkers of risk after TIA. The purpose of this study was to examine the association of both traditional and newer aPLs with outcome after TIA, in subjects without a clinical diagnosis of APS.

## Materials and Methods

### Study design

This study was a retrospective analysis of a prospectively collected cohort of patients with TIA. Subjects were enrolled within 48 h of symptom onset. To be included subjects must have had focal neurologic deficits presumed to be due to a vascular cause. Transient neurologic symptoms which were deemed to be non-vascular by the treating neurologist were excluded. The traditional time-based definition of TIA was used (symptom resolution within 24 h). Subjects were excluded if they had a terminal illness which was likely to limit their evaluation or prevent full study follow-up or if they were on warfarin with an INR ≥1.5 at the time of enrollment. Patients on warfarin were excluded by design because of the impact of long-term anticoagulation on D-dimer, a biomarker which was previously assessed in this cohort (Cucchiara et al., 2009). For each subject, a standardized case report form was completed which captured clinical information and results of diagnostic testing. Final determination of TIA mechanism and clinical events was made at 90-day follow-up. ABCD^2^ clinical risk scores were calculated and categorized as low (0–3), medium (4–5), or high (6–7) as previously described (Johnston et al., 2007). Collection of all clinical and outcome data was blinded to results of biomarker testing. A detailed description of study methods has been previously published (Cucchiara et al., 2006). Informed consent was obtained from all subjects and the study protocol was approved by the local institutional review board.

The primary outcome measure was a composite endpoint of stroke or death within 90 days, ≥50% stenosis in a vessel referable to the presenting TIA, or cardioembolic source requiring anticoagulation. The endpoint was pre-specified before patient enrollment or analysis. It was designed to identify high risk TIA patients requiring emergent evaluation and specific early interventions. In observational studies, clinical interventions may prevent high risk patients from having an event. Outcomes which focus solely on clinical events may not capture these high risk patients. The combined outcome used in this study ensures that these patients are appropriately categorized. Two secondary outcome measures were: (1) stroke or death at 90 days alone, and (2) the presence of atherosclerotic disease. Atherosclerotic disease was defined to include both clinical atherosclerosis (history of coronary artery disease/myocardial infarction, stroke, peripheral vascular disease, carotid endarterectomy, or symptomatic large-artery stenosis) and sub-clinical atherosclerosis defined by the presence of atherosclerotic plaque on imaging.

### Blood sampling and antibody testing

For all subjects peripheral venous blood was collected into sterile tubes containing 3.2% sodium citrate and then centrifuged at 1,300*g* for 10 min. Plasma was extracted and centrifuged at 10,000*g* for 3 min. Samples were frozen at −80°C until the time of analysis. Specimens were tested for aPL antibodies at a dilution of 1:101 using the following ELISA assays: QUANTA Lite^®^ anti-β2GPI IgG, IgA, IgM, anti-β2GPI-domain 4/5 IgA, anti-β2GPI-domain 1 IgG, QUANTA Lite^®^ anticardiolipin (ACA) IgG, IgA, IgM, QUANTA Lite^®^ anti-PS/PT IgG, IgM, IgG/IgM. All ELISA assays were manufactured by INOVA Diagnostics, San Diego, CA, USA and are FDA-cleared for *in vitro* use with the exception of the β2GPI-domain 1 IgG, β2GPI-domain 4/5 IgA, and the PS/PT IgG/IgM kits which are research only. All tests were performed according to the manufacturer’s instructions. Results for aCL and β2GPI were derived from five point standard curves calibrated against accepted reference standards. For all antibodies, the manufacturer’s suggested thresholds were used to define a positive test. These thresholds are determined after testing clinical samples, normal controls, disease controls, and potentially cross-reactive samples.

### Statistical analysis

Summary statistics were computed using means, medians, and counts. Groups of patients were compared with *t*-tests, Wilcoxon ranked sum, χ^2^, and Fisher’s exact test as appropriate. The primary analysis dichotomized aPL testing as positive or negative on the basis of pre-defined thresholds. We tested for an association between aPL antibodies and the composite outcome using Fisher’s exact test and univariate logistic regression. Similar analyses were conducted to evaluate the relationships between aPLs and the secondary outcomes. Antibodies which were associated with outcomes with *p* ≤ 0.10 were included in a multivariable model with ABCD^2^ score. ABCD^2^ score was treated as a categorical variable, with scores grouped into low, medium, and high risk (0–3, 4–5, and 6–7, respectively). All statistical analyses were performed using STATA version 11.0 (Stata corporation, College Station, TX, USA).

## Results

From November 2002 to June 2007, 167 subjects were enrolled. Of these, 164 subjects were available for 90 day follow-up. Of the three subjects who were not available for 90 day follow-up, one had a carotid occlusion at presentation and was categorized as endpoint positive. The other two subjects had no events or high risk mechanisms identified at hospital discharge and were categorized as endpoint negative. For nine subjects aPLs could not be measured due to missing or insufficient blood sample volume, leaving a final sample size of 158. Baseline characteristics of enrolled subjects are summarized in Table 1.

**Table 1:** **Baseline characteristics**.

	*n* (%)
Age (mean ± SD)	62 ± 15
Male sex	72 (46%)
Time from onset to enrollment, hours (mean ± SD)	26.4 ± 12.7
*Duration of symptoms*
<10 min	23 (15%)
10–59 min	42 (26%)
≥60 min	93 (59%)
*Blood Pressure at Presentation*
Systolic (mean ± SD)	153 ± 27
Diastolic (mean ± SD)	83 ± 15
*History of:*
Hypertension	103 (65%)
Diabetes	34 (22%)
CAD/MI	20 (13%)
Hyperlipidemia	63 (40%)
Prior stroke	28 (18%)
PAD	8 (5%)
Current smoker	26 (16%)
Migraine	16 (10%)
*ABCD^2^ Score*
0–3	58 (37%)
4–5	79 (50%)
6–7	21 (13%)

Overall there were 40 subjects (25%) with the composite endpoint. There were 25 subjects (15%) with >50% stenosis, 13 (8%) with cardioembolism, and 8 (5%) with clinical events. Of the clinical events five were strokes and three were deaths. There were five subjects who had both a clinical event and a high risk TIA mechanism. No subjects were clinically diagnosed with APS.

Using pre-defined thresholds to define positivity (Table 2), 16 of 158 subjects (10%) were positive for traditional aPLs (aCL or β2GPI IgG/M). There were 19 (12%) positive for aPS/PT IgM or IgG, and 26 (16%) for β2GP1 D4/5 IgA. No subjects were positive for B2GPI D1 IgG. In the univariate analysis there was no association between traditional aPLs (aCL IgA/G/M and β2GPI IgA/G/M), newer β2GPI sub-classes (D 4/5 IgA and D1 IgG), or aPS/PT IgM with either the composite endpoint or the clinical endpoint of stroke or death. Elevated aPS/PT IgG antibodies were modestly associated with the composite endpoint, although this association did not reach statistical significance (OR 4.7, 95% CI 0.8–29.2, *p* = 0.10). There was a significant association between aPS/PT IgG antibodies and the secondary endpoint of stroke or death (OR 16.3, 95% CI 2.3–116.7, *p* = 0.005). These results are summarized in Table 2. In a multivariable model (Table 3) which included ABCD^2^ score, aPS/PT IgG remained associated with stroke or death (OR 15.7, 95% CI 2.0–125.6, *p* = 0.009).

**Table 2:** **Univariate analysis**.

	Positive cut-point	# Positive (*n* = 158)	% Positive w/athero*	*p*-Value	Composite outcomeOR (95% CI)	*p*-Value	Stroke or deathOR (95% CI)	*p*-Value
aPS/PT IgG	≥30 units	5 (3%)	100	0.16	4.7 (0.8–29.2)	0.10	16.3 (2.3–116.7)	0.005
aPS/PT IgM	≥30 units	15 (9%)	67	0.79	1.1 (0.3–3.6)	0.90	3.5 (0.6–19.2)	0.15
aCL IgG	≥20 units	3 (2%)	100	0.29	6.2 (0.5–69.8)	0.14	–	–
aCL IgM	≥20 units	5 (3%)	60	1.00	0.73 (0.1–6.7)	0.78	–	–
aCL IgA	≥20 units	0 (0%)	–	–	–	–	–	–
β2GPI IgG	≥20 units	2 (1%)	100	0.53	–	–	–	–
β2GPI IgM	≥20 units	9 (6%)	67	1.00	1.5 (0.4–6.4)	0.57	2.5 (0.3–23.2)	0.41
β2GPI IgA	≥20 units	19 (12%)	68	0.47	1.1 (0.4–3.3)	0.85	1.1 (0.1–9.6)	0.92
D4/5 IgA	≥25 units	26 (16%)	58	0.66	0.9 (0.3–2.3)	0.77	0.7 (0.1–6.1)	0.76
D1 IgG	≥25 units	0 (0%)	–	–	–	–	–	–

*Legend: anti-phosphatidylserine-prothrombin (aPS/PT), β-2-glycoprotein-I (β2GPI), anticardiolipin antibodies (aCL)*.

*Percentage of subjects who were antibody positive with clinical or sub-clinical atherosclerosis.

*Blank spaces indicate no subjects who were antibody positive had the outcome of interest*.

**Table 3:** **Multivariable models, aPS/PT IgG positivity >30 units**.

	OR	(95% CI)	*p*-Value
**COMPOSITE OUTCOME**			
aPS/PT IgG	4.3	(0.7–27.8)	0.12
ABCD^2^			0.07
0–3	Ref	–	–
4–5	1.7	(0.7–4.0)	0.23
6–7	3.5	(1.2–10.6)	0.02
**STROKE OR DEATH**			
aPS/PT IgG	15.7	(2.0–125.6)	0.009
ABCD^2^			0.15
0–3	Ref	–	–
4–5	2.7	(0.3–26.1)	0.39
6–7	9.1	(0.8–99.5)	0.07

Clinical or sub-clinical atherosclerosis was present in 100 subjects (60%). APLs were not associated with atherosclerosis (Table 2).

## Discussion

Patients with TIA have a high short-term risk of stroke. Biomarkers to identify patients at high risk of subsequent stroke would assist the selection of patients for aggressive monitoring and therapeutic interventions. In the cohort of subjects with TIA examined in this study, IgG antibodies against the phosphatidylserine-prothrombin complex were associated with clinical outcome (stroke or death). The association with outcome persisted after adjustment for the ABCD^2^ score, which is the most widely used clinical risk score for predicting outcome after TIA. In contrast, traditional aPLs (aCL and β2GPI) and newer aPLs directed against the D1 and D4/5 subunits of β2GPI were not associated with outcome after TIA and no antibodies were associated with atherosclerosis.

Transient ischemic attack may be caused by a wide range of disease processes, including cardioembolism, large-artery atherosclerosis, non-atherosclerotic vasculopathy such as dissection, small artery lipohyalinosis, and hypercoagulable states. The pathophysiologic link between outcome after TIA and aPS/PT IgG is uncertain, but may be mediated by thrombosis, which is a common pathologic process underlying many TIAs. The mechanism of aPL-induced thrombosis is thought to involve antibody binding to platelets and endothelial cells with subsequent activation of the clotting cascade (Horstman et al., 2009; Cohen et al., 2010). Disruption of annexin A5 anticoagulant activity has been implicated in this process, and activation of complement may also play a role (Rand, 2000; Avalos and Tsokos, 2009). aPS/PT antibodies have previously been associated with thrombosis (Bertolaccini et al., 2005). They correlate with clinical symptoms of APS and have been previously reported in subjects with acute ischemic stroke (Atsumi et al., 2000; Okuma et al., 2006). An international task-force recently recommended a multi-center study evaluating the utility of aPS/PT as an additional marker for APS (Bertolaccini et al., 2011).

The relationship between aPLs and atherosclerosis is less well established. β2GPI is a component of atherosclerotic plaque (George et al., 1999). It has been hypothesized that autoantibodies to β2GPI contribute to the uptake of oxidized LDL by macrophages leading to the development of foam cells and plaque (Matsuura et al., 2002, 2003; Staub et al., 2006). APLs, particularly β2GPI and its D4/5 and D1 subunits, have been associated with clinical atherosclerotic disease (Iverson et al., 2006; Franck et al., 2007). Although atherosclerosis was common in antibody positive patients in the present study, we did not observe an association between any of the measured antibodies and atherosclerosis.

Some subsets of aPLs were relatively common in this cohort of TIA patients. For instance, IgA antibodies against β2GPI and the D4/5 subunit were present in a large percentage of subjects (12 and 16%, respectively). In contrast, aCL antibodies were relatively uncommon, with IgG in 2%, IgM in 3%, and IgA in 0%. This is significantly lower than the percentage of positive subjects reported in the APASS study (Anti-Phospholpid Antibodies and Subsequent thrombo-occlusive events in patients with ischemic Stroke; Levine et al., 2004). Of the 1770 subjects in APASS, 19% had aCL IgG antibodies, 5% had aCL IgM antibodies, and 10% aCL IgA antibodies. APASS did not measure β2GPI or other aPLs (Levine et al., 2004). Differences in the observed prevalence of aCL may be due to differences in the study populations. APASS was nested within a clinical trial and all participants had an ischemic stroke, rather than a TIA. APASS also used a different aCL assay (Corgenix Inc., Denver, CO, USA). Differences in specificities of the assays maybe partially responsible for the observed results. There is well documented variability across aPL assays. Inter-assay agreement is lower with aCL compared to β2GPI and lower with low-positive antibody titers, which comprised many of the positive samples in APASS (Audrain et al., 2004).

This study has several important limitations. As mentioned above, inter-assay agreement for aPLs is low. In the present study, all assays were performed with kits from the same manufacturer (INOVA Diagnostics). Our results may not be directly extrapolated to assays from other suppliers. The relatively small sample size limits power to detect associations. Although detailed clinical information is available for all subjects, the small number of clinical events limits our ability to determine whether the measured association between aPS/PT IgG and outcome is mediated by thrombosis, atherosclerosis, or some other mechanism. Furthermore, the findings should be interpreted with caution due to the small number of clinical events. There were only two patients who had both an elevated aPS/PT IgG and a clinical event. Thresholds to define positive tests were defined by the manufacturer and not specific to TIA patients. Serum was only collected at the time of the TIA. Subjects were not followed to determine if aPLs remained positive. Furthermore, a functional test of phospholipid dependent clotting was not performed as a part of the research study; although, clinically none of the subjects were suspected or diagnosed with APS. Since antibody measurements occurred after the vascular event, the possibility that aPL positivity is an epiphenomenon related to vascular occlusion or tissue injury should also be considered.

In conclusion, we found that aPS/PT IgG antibodies were associated with clinical outcome after TIA in a population of patients without APS. This association was not found with the other aPL antibodies tested. This suggests that aPS/PT IgG antibodies may be useful for risk stratifying patients after TIA; although, larger, prospective studies are needed to extend and validate these findings.

## Conflict of Interest Statement

Michael T. Mullen, MD, none; Steven R. Messé, MD, none; Scott E. Kasner, MD, has received modest honoraria for consulting from INOVA Diagnostics; Lauren Sansing, MD, none; M. Rizwan Husain, MD, none; Gary L. Norman, PhD, employee of INOVA Diagnostics, Inc.; Zakera Shums, MS, employee of INOVA Diagnostics, Inc.; Brett L. Cucchiara, MD, has received modest honoraria for consulting from INOVA Diagnostics.
